# Assessing Adherence to Preventive Behaviors Among Patients with Mental Illness in the Context of the COVID-19 Pandemic: A Scoping Review

**DOI:** 10.3390/ijerph23081001

**Published:** 2026-07-30

**Authors:** Julia Eigenheer Mühlbauer, Ana P. Ribeiro, Juliana B. de-Salles Andrade, Livi F. Testoni de Faro, Maria E. Moreira-de-Oliveira, Veronica Hühne, Carina Félix-da-Silva, Gabriela B. de Menezes, Leonardo F. Fontenelle

**Affiliations:** 1 Obsessive, Compulsive, and Anxiety Spectrum Research Program, Institute of Psychiatry of the Federal University of Rio de Janeiro (UFRJ), Av. Venceslau Brás, 71, Botafogo, Rio de Janeiro 22290-140, RJ, Brazil; juliaeigenheer@ufrj.br (J.E.M.); anapaulapsr@gmail.com (A.P.R.); livifaro@gmail.com (L.F.T.d.F.); enfcarinafelix@gmail.com (C.F.-d.-S.);; 2 D’Or Institute for Research and Education (IDOR), Rio de Janeiro 22281-110, RJ, Brazil

**Keywords:** COVID-19 pandemic, preventive behaviour, mental disorder

## Abstract

**Highlights:**

**Public health relevance—How does this work relate to a public health issue?**

- Individuals with mental disorders represent a vulnerable population during infectious disease outbreaks, often showing lower adherence to COVID-19 vaccination and preventive measures, which may increase infection risk and adverse outcomes.
- Understanding barriers to preventive health behaviors in this group is essential for reducing health inequities and improving pandemic preparedness and response.

**Public health significance—Why is this work of significance to public health?**

- This review synthesizes evidence from 50 studies and highlights that people with severe mental illnesses frequently exhibit lower vaccination uptake compared with the general population and other control groups, leading to higher hospitalization rates and greater utilization of public healthcare resources.
- The findings reveal a major gap in the literature, with very few intervention studies designed to improve adherence to preventive behaviors despite evidence suggesting that such interventions can be effective. The available evidence may help guide the development of targeted public health policies and strategies to enhance adherence to preventive measures and improve health outcomes in this vulnerable population.

**Public health implications—What are the key implications or messages for practitioners, policy makers and/or researchers in public health?**

- Public health strategies should prioritize tailored outreach, psychoeducation, and accessible preventive care programs to improve vaccination coverage and adherence to protective behaviors among people with mental disorders.
- Researchers and policy makers should invest in the development and evaluation of targeted behavioral interventions, particularly for individuals with severe mental illnesses, to strengthen resilience during future public health emergencies.

**Abstract:**

Background: The COVID-19 pandemic has become a global public health emergency, causing substantial disruptions in societal norms and behaviors. Among the affected population, individuals with mental health disorders are particularly vulnerable, facing heightened infection rates and worse outcomes. These challenges can be linked to factors such as insufficient adherence to vaccination and other preventive measures. Objective: This study aims to investigate the literature on the adherence of individuals with mental disorders to vaccination and other preventive behaviors such as social distancing, use of face masks, and hygiene practices during the COVID-19 pandemic. Methods: This scoping review was performed following the methodological guidelines outlined by the Joanna Briggs Institute and reported in accordance with the Preferred Reporting Items for Systematic Reviews and Meta-Analyses extension for Scoping Reviews checklist. A comprehensive search for published research containing original data was conducted in four databases (Embase, Medline, PsycINFO, and Web of Science). Results: The search yielded 2778 records, and 10 additional relevant papers were identified through handsearching, resulting in a total of 50 studies meeting all inclusion criteria. Most of them focused on vaccination adherence, with approximately half reporting lower adherence in the patient group, particularly among individuals with severe mental illnesses (SMIs), compared to the general population or to other control groups. However, some studies reported comparable—or, in specific psychiatric populations, even higher—levels of adherence to preventive behaviors. Out of the fifty selected studies, only four employed an interventional approach aimed at increasing preventative behaviors, but they were effective in most of the studies. Conclusions: This review underscores a noticeable lack of intervention studies employing psychoeducational strategies to mitigate risky behaviors among individuals with mental disorders. This gap emphasizes the urgent need for increased research focus in this area, as it holds promise for reshaping future approaches and strategies to address other crises within this vulnerable population.

## 1. Introduction

Coronavirus disease 2019 (COVID-19) appeared in late 2019 and rapidly evolved into one of the most significant public health challenges of the twenty-first century [1]. As the outbreak escalated, the World Health Organization (WHO) declared COVID-19 a pandemic on 11 March 2020, recognizing its unprecedented global public health impact [2]. Since then, more than 775 million cases and over 7 million deaths have been reported globally [3]. Beyond its direct health consequences, the pandemic profoundly disrupted social norms and daily life, generating lasting concerns related to infection, mortality, and long-term health outcomes, while also contributing to widespread psychosocial distress across populations [4].

Despite the WHO’s declaration in May 2023 that COVID-19 no longer qualifies as a public health emergency of international concern (due to reduced hospitalization and mortality rate) [5], and the increased immunity achieved through over 13 billion vaccine doses administered as of December 2023, the effective long-term management of the disease remains a formidable challenge [6]. A recent update from the WHO, published in January 2024, underscores that COVID-19 continues to impact people’s lives and national health systems, with thousands of individuals still succumbing to the disease and approximately 6% of symptomatic infections leading to post-COVID-19 conditions. Nevertheless, after the initiation of the immunization campaign in December 2020, there has been a significant decline in the global vaccination rate. In the second semester of 2023, only 59 million doses were administered, a stark contrast to the 327 million doses administered six months earlier. This decline is observed despite ongoing recommendations to continue vaccinating, especially for high-risk groups [7].

Because COVID-19 is primarily transmitted through respiratory droplets released during close interpersonal contact [2,8], and because the disease can present with a wide range of clinical manifestations, from asymptomatic infection to severe illness with multiple complications [9], preventive measures became a central component of the public health response from the earliest stages of the pandemic. These measures included physical distancing, reinforcement of personal hygiene practices [2], the use of face masks and other protective equipment [8], home confinement, contact tracing, and temporary restrictions on access to public spaces [9]. Although vaccination has substantially changed the course of the pandemic, several preventive recommendations remain relevant, including mask use when symptomatic, regular hand hygiene, and self-isolation following symptoms or a positive test result [7,10].

People with mental health disorders represent one of the groups most vulnerable to the negative consequences of the COVID-19 pandemic [11,12,13]. Beyond the well-established association between mental illness and reduced life expectancy [14], evidence suggests that individuals with mental disorders experienced higher rates of COVID-19 infection during the pandemic [15,16], together with an increased risk of mortality [17,18]. In addition, lower adherence to vaccination and other preventive health behaviors has historically been reported in this population [19], further increasing the challenges associated with protecting their health during infectious disease outbreaks.

Therefore, it is evident that healthcare providers must recognize the enduring and pronounced vulnerability experienced by individuals with mental illness and the deepening of pre-existing health disparities, notably exacerbated during crises such as the COVID-19 pandemic. According to the WHO, prior to the onset of the pandemic, 970 million people globally, equating to one in every eight individuals, were living with a mental disorder. These numbers saw a significant increase following the pandemic, exemplified by a notable 28% rise in cases of major depressive disorder [20]. Although research on vaccine hesitancy (VH) has expanded in the general population and within groups of individuals with mental disorders, studies investigating other preventative behaviors (e.g., adherence to mask use, social distancing, and handwashing) are less consistently explored.

This scoping review aims to map and synthesize the available evidence on adherence to vaccination and other preventive behaviors among individuals with mental disorders during the COVID-19 pandemic, while also identifying factors that may influence these behaviors. A comprehensive understanding of these patterns may help inform strategies designed to improve the uptake of preventive measures, reduce health risks, and address barriers faced by this vulnerable population. Furthermore, the findings may provide valuable insights for healthcare professionals, researchers, and policy makers seeking to strengthen preparedness and response efforts in future public health emergencies.

## 2. Materials and Methods

**Figure 1:**
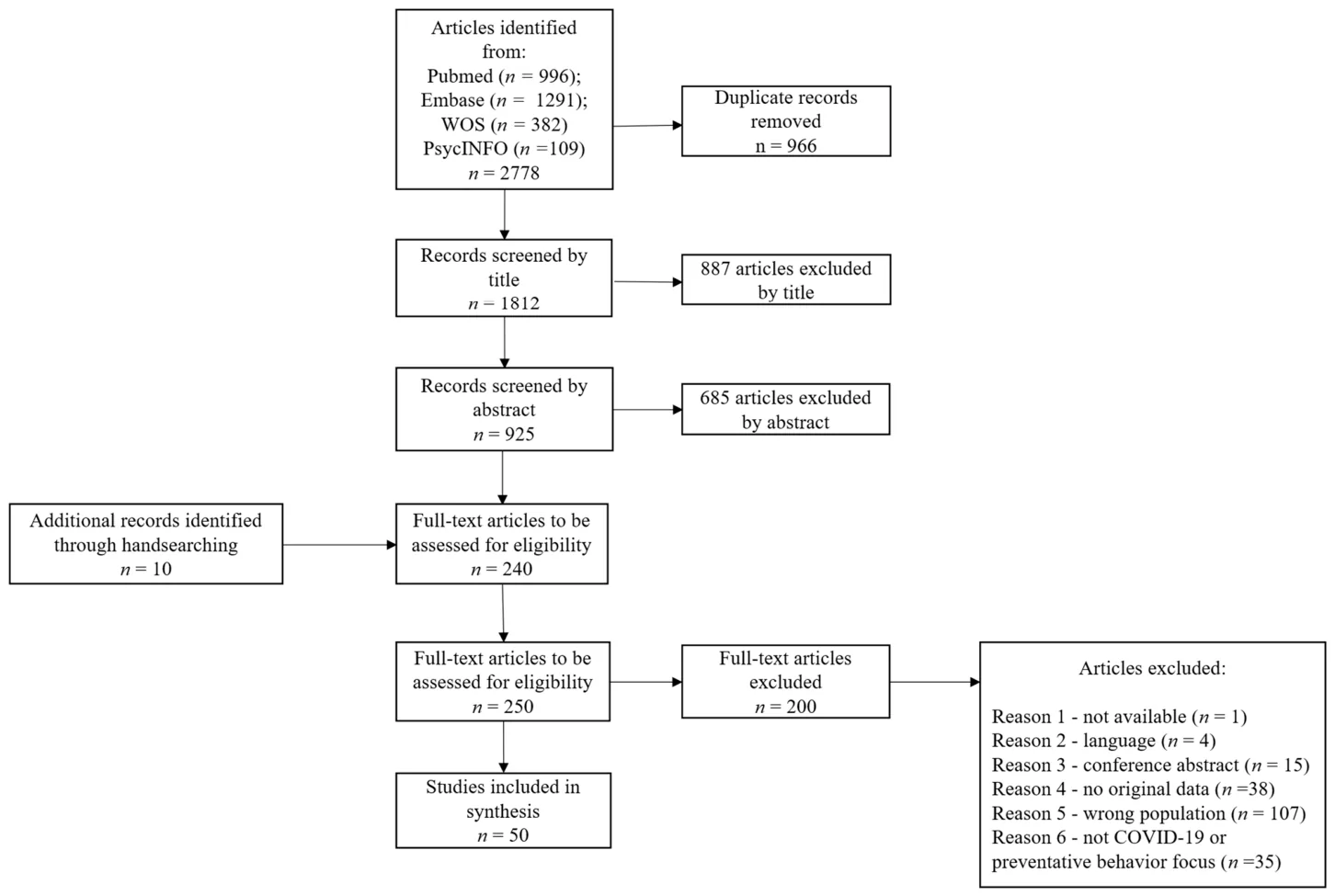
PRISMA flow chart for study selection.

This scoping review was conducted in accordance with the methodological framework proposed by the Joanna Briggs Institute (JBI) for scoping reviews [21] and is reported following the Preferred Reporting Items for Systematic Reviews and Meta-Analyses extension for Scoping Reviews (PRISMA-ScR) guidelines [22]. To report the study selection process, an adapted version of the PRISMA flow diagram is provided in Figure 1. The diagram provides a comprehensive overview of the numbers and rationale behind the exclusion of studies, as per the recommendations outlined in the PRISMA-ScR checklist. Table S1 in the Supplementary Materials presents the PRISMA-ScR checklist for this scoping review [22].

The review protocol was previously registered at the Open Science Framework (OSF) and is retrievable in a designated DOI (10.17605/OSF.IO/STKBV). Furthermore, the protocol underwent publication in *Clinical Neuropsychiatry* in 2023, with a DOI assigned for reference (10.36131/cnfioritieditore20230408).

### 2.1. Search Strategy

In accordance with the guidelines stipulated by the Joanna Briggs Institute (JBI), a meticulous three-step search strategy was implemented. Initially, a constrained search was conducted on two online databases, namely MEDLINE and Web of Science. The objective was to identify pertinent text words in titles and abstracts, and index terms employed in the articles. Subsequently, a second search encompassed four electronic databases—MEDLINE/PubMed, Embase, Web of Science, and PsycINFO—employing the identified text words and index terms. Only studies in English, Spanish, or Portuguese were considered as a matter of practical feasibility.

Lastly, an exhaustive review of the reference lists of the incorporated studies and other relevant papers was undertaken to identify additional sources. A comprehensive outline detailing the search strategy deployed in each database is provided in Table 1 below.

### 2.2. Study Selection

Duplicate titles identified across databases were initially excluded through a reference manager tool (Endnote^®^), followed by a meticulous manual search for further confirmation. The study selection process comprised two sequential screening stages using the Rayyan tool. Initially, titles underwent independent evaluation by two authors, and the same rigorous screening process was applied to abstracts, determining their eligibility for a comprehensive full-text reading based on predefined inclusion criteria. The final stage involved a thorough assessment of full-text articles by two authors to ascertain adherence to the inclusion criteria. In instances of disagreements, resolution was sought through consultation with a senior expert from the research team (LF). Our search and selection process specifically targeted peer-reviewed studies reporting original data across diverse study designs, excluding reviews due to their lack of new primary data.

The inclusion criteria, as well as all methodological procedures adopted in this scoping review, were predefined in the published study protocol, which has been previously cited in this manuscript. The inclusion criteria were as follows:

- Publications written in English, Spanish, or Portuguese.
- Publications released between January 2020 and October 2023 to align with the timeframe of the pandemic.
- Studies focusing on adherence to vaccination and/or other preventive behaviors, such as social isolation, mask usage, and hygiene protocols.
- Studies conducted within the context of the COVID-19 pandemic.
- Samples comprising patients diagnosed with at least one psychiatric disorder listed in the Diagnostic and Statistical Manual of Mental Disorders (DSM) and/or the International Classification of Diseases (ICD).

### 2.3. Data Extraction

To ensure a systematic and thorough extraction of data, two researchers used a spreadsheet to document essential information. This information encompassed the first author, country of origin, publication date, study design, recruitment setting, sample size, patients’ profile (including diagnoses and outpatient or inpatient status), demographics, key findings pertinent to the scoping review topic, and main conclusions. Throughout this phase, one reviewer conducted the data extraction, while the first author oversaw the process to ensure the accuracy and completeness of the extracted information.

### 2.4. Data Synthesis

Following data extraction, the evidence was synthesized descriptively. Studies were grouped according to study design and diagnostic category, and the findings were summarized narratively to identify recurring patterns, similarities, and differences across the included studies.

## 3. Results

### 3.1. Search Findings

The search yielded 2778 records, from which 1812 articles remained after duplicates were removed. A meticulous screening process ensued, commencing with the evaluation of titles for all articles, followed by a review of 925 abstracts. This process narrowed down the selection to 240 full-text articles, subjected to further analysis for eligibility based on predefined inclusion criteria outlined in the methods. An additional 10 relevant papers were identified by examining the reference lists for additional sources.

Subsequently, each of the 250 papers underwent a comprehensive full-text review. During this phase, exclusion criteria were applied to eliminate articles that did not recruit samples with well-defined psychiatric disorders listed in the DSM or the ICD (*n* = 107 records), did not concentrate on adherence to preventive behaviors in the context of the COVID-19 pandemic (*n* = 35 records), did not report original data (*n* = 38 records), were not published in English, Portuguese, or Spanish (*n* = 4 records), constituted conference abstracts (*n* = 15 records), or had unavailable full texts (*n* = 1 record).

Ultimately, 50 publications fulfilled all inclusion criteria. Notably, three studies conducted by the same research group, utilizing the same database but with distinct sample sizes, periods, and outcomes, were treated individually in the descriptive analysis (Figure 1).

### 3.2. Study Characteristics

Our findings are summarized in six tables (Table 2, Table 3, Table 4, Table 5, Table 6 and Table 7), including essential information from the 50 selected articles. This information encompasses details such as the first author and publication year, country of origin, study design, recruitment setting, sample size, patients’ psychiatric diagnoses, and key conclusions relevant to the scoping review topic. For the organization of the six tables, the studies were broadly classified into two main categories: qualitative and quantitative. The quantitative studies were further subdivided based on differences in study design and sample diagnosis.

The initial subdivision focused on cross-sectional studies with samples of patients with neurodevelopmental disorders, specifically autism spectrum disorder (ASD) and attention deficit and hyperactivity disorder (ADHD). The second subdivision centered on cross-sectional studies with patients diagnosed with severe mental illness (SMI). This category included samples with severe mental disorders like schizophrenia (SCZ), bipolar disorder (BD), and major depressive disorder (MDD), among others. The third subdivision comprised studies with mixed samples, incorporating a range of disorders that did not fall, at least not always, under the SMI category, such as anxiety disorders and OCD. Another classification within the quantitative studies involves longitudinal studies, which could be either retrospective or prospective. The final category consists solely of interventional studies.

Out of the 50 selected studies, the majority predominantly focused on vaccination adherence (VA) (*n* = 39, 78%), with a smaller proportion addressing general preventive behaviors such as social distancing, mask use, and hygiene measures (*n* = 11, 22%). In terms of geographical distribution, a significant portion of the studies were performed in Europe (*n* = 28, 24%), followed by the US (*n* = 11, 22%), Israel (*n* = 6, 12%), and China (*n* = 6, 12%). Four studies originated from Turkey (8%), three (6%) from India, and one (2%) each from Australia, Qatar, Japan, and Korea.

Two studies (4%) were conducted simultaneously in three different countries, Western and non-Western. Regarding the publication year, most were published in 2022 (*n* = 22, 44%), followed by 2021 (*n* = 19, 38%), 2023 (*n* = 7, 14%), and finally 2020 (*n* = 2, 4%). In the analysis of the diagnostic samples in the studies, 19 (39%) had mixed samples, 15 (30%) involved patients with severe mental illness (SMI), 10 (20%) included only schizophrenia or other psychotic disorders, three (6%) included only ASD cases, two (4%) involved only ADHD, and one (2%) included only opioid use disorder (OUD).

Among the studies that compared the rates of vaccine adherence (VA) and compliance with the general preventative measures between patients and the general population (*n* = 32, 64%), half (*n* = 16) reported lower adherence/compliance in the patient group, and few (*n* = 3) indicated higher adherence/compliance among patients compared to the general population or control group. It is noteworthy that among these three studies, one involved an ASD sample [55], one included patients with ADHD [57], and one comprised a mixed diagnosis, including major depressive disorder and anxiety disorders, where patients were more likely to pay for the COVID-19 vaccine than controls [26].

### 3.3. Vaccine Adherence

#### 3.3.1. Decrease

A matched, controlled, retrospective cohort study assessed first, second, and booster vaccinations among 34,797 individuals with schizophrenia and 34,797 matched controls. Individuals with schizophrenia were significantly less likely to be vaccinated with the first vaccine, with 20.7% unvaccinated in the schizophrenia group compared with 14.5% unvaccinated in the control group. Once vaccinated, no significant differences were observed between the groups in the uptake of the second vaccine. Of those receiving the second vaccination, significant differences were again found between the groups, with 74.7% completing the third (booster) vaccination in the schizophrenia group, compared with 77.9% in the control group. Time to reach vaccination was significantly longer for the group with schizophrenia but primarily with the first vaccine and to a smaller extent with the booster vaccine [65]. In an earlier study conducted by the same authors, the disparity in vaccination rate between the patient and control groups was particularly evident among individuals aged 60 and above, as well as among male participants [40].

In a comparative analysis of adherence levels among diverse psychiatric illnesses, a study encompassing 14,365 patients revealed a higher prevalence of VH across all examined psychiatric comorbidities, except for alcohol use disorder (AUD). Following adjustments for sociodemographic characteristics and physical health conditions, substance use disorders (SUD) and tobacco use/nicotine dependence demonstrated a significant positive association with heightened odds of vaccine hesitancy, whereas BD exhibited a significant inverse association with VH, suggesting a distinctive pattern of attitudes toward vaccination within this specific psychiatric cohort [33]. In a later study, vaccine hesitancy exhibited lower prevalence across the majority of assessed physical comorbidities.

The factors contributing to vaccine hesitancy include a range of commonly cited reasons that are also prevalent in the general population [73,74]. Primary concerns include apprehensions about adverse effects and vaccine safety [24,25,26,30,36,69], concerns about potential exacerbation of mental disorders or side effects of psychotropic drugs [36], worries about the rapid development of the COVID-19 vaccines [25,69], uncertainties about the effectiveness [25,36] or the necessity of the immunization [69], the absence of specific clinical guidelines for vaccination in people with mental disorders [27], and the stigma surrounding mental disorders [45].

Few studies have also investigated whether people with mental disorders were more prone to belief in conspiracy-based beliefs regarding the vaccination for COVID-19.

Accordingly, the endorsement of conspiracy-based beliefs correlated with the presence of psychiatric symptomatology [25,69,70], particularly positive [70] and cognitive symptoms [27,48]. In the same vein, vaccine-hesitant patients displayed greater severity of “paranoia”, assessed by the Paranoia subscale of the Specific Psychotic Experiences Questionnaire (SPEQ-P) [64], and psychotic symptoms [48], whereas lower vaccination rates were observed in hospitalized individuals with mental disorders [36]. Interestingly, despite exhibiting higher confidence in the benefits of vaccination, individuals with schizophrenia had vaccination rates that were both lower than those of a control group and not significantly associated with global cognitive functioning, psychotic symptoms, or paranoid ideation, implying the presence of structural impediments rather than attitudinal barriers when it comes to getting vaccinated within this clinical population [50].

#### 3.3.2. Increase

Despite the evidence suggesting vaccine hesitancy to be particularly common in people with mental disorders, there are also factors that may increase VA among individuals with mental disorders. For instance, the provision of convenient vaccination opportunities, particularly when offered by healthcare providers [30] or in familiar locations [25], and high confidence in the public healthcare system and recommendations from the government [26] have been found to enhance vaccine uptake. Other facilitating factors include a perceived high risk of mortality, financial strains due to the pandemic, beliefs in the preventive efficacy of vaccines, confidence in their safety, and a good insight into one’s mental disorder [27]. Increased vaccine acceptance has also been associated with consistent mask-wearing in people attending an SUD clinic [43] and the presence of medical comorbidities such as diabetes, hypertension, obesity, or ischemic heart disease in individuals with schizophrenia [59].

Demographic factors may also increase COVID-19 vaccine uptake, with older individuals [33,38,43,61], highly educated people [33], and urban residents [36] with mental disorders more likely to get vaccinated. Moreover, residing in psychosocial centers [61] and recent utilization of inpatient or outpatient mental healthcare [37] have been correlated with a positive vaccination status. Regular recipients of the influenza vaccine and those who trust formal information sources are also more inclined to accept COVID-19 vaccination [38]. Notably, individuals with higher levels of gratitude and agreeableness, coupled with lower levels of depression, are predictive of greater vaccine acceptance [38].

Interestingly, in two studies exclusively focusing on ADHD samples, one found no significant difference between patients and controls in terms of COVID-19 infection status, vaccination adherence, and perception of COVID-19 as dangerous [56]. In the other study, a large sample of adolescents diagnosed with ADHD showed a higher COVID-19 vaccination rate compared to the non-ADHD group, with females being particularly more likely to be vaccinated [57]. It is unclear whether these rates were influenced by parental beliefs and attitudes regarding vaccination. However, in another study, increased age, the presence of anxiety disorders, a lower level of parental vaccine hesitancy, the existence of a chronic disease in a family member, and a family member’s death due to COVID-19, along with positive parental vaccination status, were identified as predictors of adolescent vaccination [32].

### 3.4. Compliance with General Preventive Measures

#### 3.4.1. Decrease

Most studies describing less compliance with general preventive measures were reported in people with severe mental illnesses (SMIs). In a Japanese cross-sectional study involving a mixed sample of 1166 patients from a psychiatric hospital and one mental health clinic, patients with ICD-10 schizophrenia, schizotypal, and delusional disorders reported keeping the 3Cs to be avoided (closed spaces, crowded places, and close-contact settings) less frequently in their minds, using, changing, and washing their masks more rarely, and washing their hands and gargling less frequently. They also reported lower participation in online shopping and online meetings than patients in other diagnostic categories [34]. Although these activities are not preventive behaviors themselves, this finding may reflect lower engagement with digital resources and online social interactions during the pandemic.

Similar findings were reported in a Spanish study assessing a mixed sample of 185 patients with SMI and 85 patients with less severe mental disorders [63]. In this study, the proportion of patients who consistently wore a mask was lower in the SMI group, although this difference did not reach statistical significance. Conversely, a significantly lower percentage of individuals in the SMI group reported “always” engaging in washing and social distancing compared to the less severe group of patients [63].

An Indian cross-sectional telephone survey with 132 stable individuals with SMI may help explain these findings [39]. In this study, 73.5% of patients were not worried about getting COVID-19 and lacked adequate knowledge to identify symptoms and only 36% were aware of precautionary strategies against COVID-19. Alarmingly, 8.3% of participants were completely unaware of the pandemic. In multivariate regression analysis, patients with lower socioeconomic status, low literacy levels, or inadequate social support showed less knowledge related to COVID-19 [39].

Not surprisingly, a study investigating how the parents of 87 individuals with ASD thought their children responded to COVID-19 reported similarly concerning results [54]. In this study, most of the sample had problems understanding what COVID-19 represents and the preventative measures it requires. Parents also had challenges implementing social distancing and other hygienic measures [54].

#### 3.4.2. Increase

People with less severe mental disorders tended to be overrepresented among individuals who exhibit an “increased” compliance with preventative measures against COVID-19. For instance, in a mixed sample encompassing Turkish patients with anxiety disorders, depressive disorders, adaptation disorders and OCD, no statistically significant differences were found in the total cleanliness, social distancing, and hygiene scores compared to healthy individuals. However, the patient group exhibited higher scores in mask-wearing compared to the control group, while the control group demonstrated increased scores in food and beverage hygiene [67]. Additionally, individuals with affective disorders were more likely to use face masks, to stay home, to work remotely and to avoid public spaces/transportation than patients with schizophrenia spectrum illnesses in an American study investigating the prevalence of engagement in COVID-19 preventative behaviors among individuals with SMI [44]. Despite reporting more psychological distress, most individuals with SMI reported performing multiple behaviors intended to prevent COVID-19 infection, suggesting a good level of awareness of COVID-19 among patients with severe but stable mental illness.

Similar findings were reported in the Japanese study including 1166 outpatients reported above, where the mood disorder group exhibited a notable reduction in time spent outside, while patients with neurotic, stress-related, and somatoform disorders were stricter in terms of mask management than patients with SMI [34]. Although it is tempting to ascribe increased compliance with general preventive measures to abnormally increased health anxiety, contamination fears, compulsive rituals or avoidant behavior, we were unable to find studies linking these symptoms to the “darker” side of increased compliance. Certainly, throughout the pandemic, clinicians often reported problems differentiating “good” from “bad” compliance [75].

It is also unclear whether certain subgroups of patients with SMI may report not decreased but normal or increased engagement with preventative general measures against COVID-19. For instance, one qualitative study suggested that patients with schizophrenia had similar attitudes toward lockdown to the general population [23]. Another Turkish study reported that 88.2% and 89.5% of their sample of patients with schizophrenia reported using masks and engaging in social distancing, respectively [41]. It is possible, however, that some patients exhibit delusional concerns regarding COVID-19 that may promote mask use or asociality that may facilitate social distancing. For instance, individuals at clinical high risk (CHR) for psychosis reported that fear of contracting COVID-19 kept them from enjoying recreational activities/hobbies, pursuing goals, and socializing more than healthy controls. In addition, asociality (but not other symptoms) correlated positively with greater adherence to COVID safety precautions [42].

### 3.5. Interventional Studies

In the context of the reviewed subject, only four interventional studies meeting the inclusion criteria were identified [69,70,71,72]. Of these, three studies exclusively focused on individuals with SMI [69,70,72], while one study included a mixed sample comprising individuals with both SMI and non-SMI disorders, such as ADHD, anxiety and adjustment disorder [71]. A total of 607 patients participated in these interventions. All studies focused on testing interventions to increase vaccination rates among patients [69,70,71], but only one also tested an intervention to enhance patients’ functioning and resilience in coping with the COVID-19 pandemic [72]. One study had “negative” results [70], and the remaining three had “positive” findings.

The “negative” study was a non-randomized trial comparing two mobile mental health units (MMHU) providing care to individuals with SMIs [70] in two different research sites. One site adopted a proactive approach to patient vaccination by informing patients of the benefits of the vaccination, while the other maintained a neutral approach, with no discussion or encouragement regarding vaccination. The study found no statistically significant differences in vaccination rates among patients attending the two units or among patients with schizophrenia spectrum disorders or bipolar disorder [70].

On the other hand, the remaining three interventional studies demonstrated a favorable impact of psychoeducational programs on vaccination adherence among patients [69,71,72]. In a cohort of 193 outpatients with SMI undergoing clozapine treatment, active involvement of psychiatric providers led to an improved vaccination rate [69]. Similarly, Ref. [71] demonstrated positive outcomes in a sample of 115 patients with diverse mental disorders, such as anxiety, depression and psychotic disorders, who received an educational intervention delivered by previously trained mental health therapists. Finally, Veltro et al. conducted a 1-year study involving 37 SMI patients trained with the InteGRO approach, a salutogenic–psychoeducational recovery-oriented approach based on four modules: definition of goals, effective communication, emotional perception and problem-solving. Their findings indicated an improvement in coping with problems and unpredictable events, as well as an increase in personal and social functioning, including vaccination adherence. None of the three studies included a comparison group; thus, they were unable to establish causation or determine whether patients’ vaccination status was influenced by the intervention or other unknown external factors.

## 4. Discussion

In this scoping review, we identified 50 studies examining VA and compliance with general preventative behaviors among patients with mental health disorders during the COVID-19 pandemic, published between 2020 and 1 October 2023. Notably, 82% of these studies were published between 2021 and 2022, with nearly 80% specifically addressing vaccination adherence. Out of the 50 studies, 33 had a cross-sectional design, highlighting a lack of longitudinal research in this area. In general, studies (mostly cross-sectional) reported decreased vaccine uptake and poor compliance with general preventative measures among patients with SMI (e.g., schizophrenia), although individuals with less severe mental disorders (e.g., mood, anxiety and obsessive–compulsive-related disorders) may exhibit higher social distancing and mask use. Also, among the four included interventional studies, a positive effect of psychoeducational programs for increasing vaccination adherence among patients with mental disorders was observed in three. The significance of these findings is underscored by the elevated COVID-19-related morbidity and mortality consistently reported among people with mental disorders. The evidence synthesized in this review suggests that lower vaccination uptake and poorer adherence to preventive behaviors observed in specific psychiatric populations may contribute to this increased vulnerability, alongside biological, clinical, and social determinants previously described in the literature. These findings provide an important context for understanding the disproportionate impact of COVID-19 on people with mental disorders.

Importantly, lower adherence to preventive behaviors was not consistently observed across all psychiatric populations. Some studies reported satisfactory awareness of COVID-19 and good adherence to preventive measures among individuals with severe mental illness, particularly those receiving regular psychiatric care [1,44]. Moreover, interventional studies showed that active involvement of mental health professionals and psychoeducational approaches were associated with improved vaccination uptake and functioning [69,72]. Together, these findings suggest that continuous psychiatric care and appropriate treatment may contribute to better engagement with preventive behaviors during public health emergencies.

### 4.1. Increased Mortality Rates Among People with Mental Disorders

It is widely recognized that the global pandemic of COVID-19 had an unparalleled impact on populations worldwide. However, individuals with mental disorders experienced a disproportionate burden [4,17,18,76]. A comprehensive study, analyzing a nationwide database of electronic health records of 61 million adult patients from 360 American hospitals and 317,000 providers, revealed that recent diagnoses of mental disorders, including ADHD, bipolar disorder, depression, and schizophrenia, significantly increased the risk of COVID-19 infection. Notably, the strongest effects were observed for depression and schizophrenia. Moreover, individuals with both a recent mental disorder diagnosis and COVID-19 infection exhibited a higher death rate of 8.5%, compared to 4.7% among COVID-19 patients without a mental disorder [4].

Accordingly, in a retrospective cohort study involving 7348 adults with confirmed COVID-19 infection, a diagnosis within the schizophrenia spectrum emerged as a significant risk factor for death, ranking second only to age in the strength of association among various demographic and medical risk factors examined in the sample [17]. A Korean study also identified a positive correlation between SMI and severe clinical outcomes in COVID-19 cases [1]. Admittedly, an increased risk of developing infection and complications in individuals with mental disorders is not limited to COVID-19 [13]. For instance, in a British study, people admitted to hospital for schizophrenia, bipolar disorder, depression or anxiety had an increased risk of subsequent pneumococcal disease, and the risk was associated with the psychiatric disorder rather than with the event of hospitalization as it remained high for years after discharge [77].

### 4.2. Risk Factors for Infection and Death Among People with Mental Disorders

Several risk factors may contribute to increased infection rates and worse prognoses in this population. These factors include high rates of obesity [78], metabolic syndrome [79,80], cardiovascular disease [81,82,83], and side effects from psychotropic medications [79,84,85]. Indeed, many individuals experiencing mental illness often require regular use of antipsychotic, antidepressant, and mood-stabilizing medications, which are frequently associated with substantial weight gain, obesity and related cardiometabolic abnormalities [12]. Concurrently, cardiovascular disease has been identified as a risk factor not just for infection and death but also for a higher incidence and severity of so-called Long COVID [86].

Other relevant risk factors include physical circumstances like residential instability [87], homelessness [88,89] and decreased social support [90,91]. Cognitive impairment [92,93], disorganized behavior [94] and poor insight [93] also play a role. Compared with the general population, the prevalence of smoking [95], alcohol and other substance abuse [96] is higher in patients with mental disorders. It is crucial to note that, particularly in the context of COVID-19 infection, fatalities are associated with comorbidities in up to 90% of cases, most commonly involving cardiovascular diseases, diabetes, hypertension and chronic obstructive pulmonary conditions [97].

### 4.3. Increased Infection and Mortality Rates May Reflect Poor Adherence to Preventive Behaviors Among People with Mental Disorders

Some of the factors associated with increased infection and mortality rates among people with mental disorders could also account for the noted lower adherence to preventive behaviors in various studies included in this review, as presented in Table 2, Table 3, Table 4, Table 5, Table 6 and Table 7. For instance, individuals with SMI commonly experience limited knowledge of physical illnesses, and may lack understanding of preventive behaviors [98]. Consequently, they may face greater challenges in complying with protective hygiene measures, social distancing, and other health guidance related to the COVID-19 pandemic. The association between the occurrence of mental disorders and poor adherence to preventive behaviors is not new and has been reported in the context of other (non-COVID-19) infections.

For instance, a study conducted in an outpatient psychiatric clinic in Alabama revealed a lower rate of flu vaccination among individuals with mental illness compared to those without mental illness [99]. Additionally, a systematic review encompassing 52 studies has underscored the presence of highly risky behaviors associated with HIV transmission, such as unprotected intercourse and multiple sexual partners, among adults with severe mental illness [100]. Positive predictors of willingness to adopt protective actions were identified in a study conducted before the coronavirus outbreak, specifically during an influenza pandemic with individuals diagnosed with schizophrenia. These predictors included self-efficacy, perceived likelihood of contracting the infection, higher educational status, and overall perceived risk from the flu [101].

Unfortunately, it may have been almost impossible for certain groups of people with mental disorders to follow the stricter stay-at-home orders and social distancing recommended during the COVID-19 pandemic. For instance, specific life circumstances of individuals with psychiatry conditions often compel them to reside in crowded and communal places, such as hospitals, shelters, psychiatric units, residences, and prisons [76]. These settings present heightened risks for the rapid dissemination of infections, making it difficult for individuals to quarantine effectively [13]. Additionally, fear of stigma of mental illness and discrimination in healthcare can also contribute to reluctance and low rates of help-seeking among individuals with mental disorders who become infected with COVID-19 [102].

### 4.4. Interventions to Increase Adherence to Anti-COVID-19 Measures

Out of the fifty studies included, only four adopted an interventional approach to promote preventive behaviors during the COVID-19 pandemic, particularly focusing on VA. Based on the findings of three out of the four studies [69,71,72], psychoeducational programs and active involvement from healthcare providers appear to be promising strategies for improving adherence to preventive healthcare among people with mental disorders. Although psychoeducation is broadly beneficial and applicable to many populations, these findings highlight its potential value for addressing barriers specific to this vulnerable group.

These strategies may be effective not only in increasing adherence to preventive behaviors like vaccination but also in helping patients cope with traumatic events and improve their quality of life, even with patients who may not initially seem open to the information. Nevertheless, it is crucial to acknowledge certain limitations within the studies, notably the absence of a control group in all of them, which impedes the establishment of causation, small sample sizes or a lack of representativeness, potentially limiting the generalizability of findings to the broader population with mental illness. We were unable to identify studies testing strategies aimed at decreasing excessive preventative behaviors (e.g., excessive social isolation or handwashing) among people suffering from conditions such as obsessive–compulsive disorder (OCD), generalized anxiety disorder (GAD) and illness phobia, among others.

## 5. Limitations

Our study has some notable limitations. Firstly, the scope of the review was limited to indexed English-, Portuguese-, or Spanish-language peer-reviewed studies focusing on preventive behaviors in a population with mental disorders during the COVID-19 pandemic. Although language limitation prioritized feasibility, the COVID-19 pandemic affected the world as a whole and it is likely that important studies performed in other languages were missing. Additionally, it is important to acknowledge that the selected articles varied in content, sample size, and methodology. Most studies employed a cross-sectional design primarily addressing vaccine adherence among patients with multiple diagnoses, complicating the ability to observe differences between various conditions. Finally, it is important to highlight that the lack of comparable outcomes across the investigations prevented the possibility of conducting a meta-analysis or a more circumscribed systematic review. Yet, despite the above-mentioned limitations, we feel we were able to cover a substantial fraction of the literature, as only one identified article remained untraceable despite our best efforts to contact its authors.

## 6. Conclusions and Future Directions

The outcomes of this review highlight a scarcity of intervention studies employing psychoeducational strategies to mitigate risky behaviors among individuals with mental disorders when compared to the abundance of observational studies. This research gap underscores the imperative for increased focus on interventional research in this field, as it has the potential to reshape future directions and strategies for addressing other crises within this marginalized population. Furthermore, gaining insights into the phenomenon of low preventive behavior rates in patients with mental illness could provide valuable guidance for mental health professionals and policy makers, facilitating efforts to eliminate behavior disparities in this vulnerable population.

## Figures and Tables

**Table 1:** Search strategy—from 1 January 2020 to 1 October 2023.

Database	Field and Filters	Round	Search Terms	Combinations (Round Numbers)
MEDLINE/ PUBMED 996 results	All Fields English, Portuguese, Spanish, from 1 January 2020 to 1 October 2023	1	SARS-CoV-2 OR coronavirus OR COVID-19 OR pandemic	1 AND 2 AND 3
		2	(vaccin* OR immunization OR mask OR quarantine OR lockdown OR “social distancing” OR “social isolation” OR isolation OR hygiene) AND (hesitan* OR accept* OR reject* OR uptake OR adheren* OR refus*)	
		3	(patient* OR client*) AND (psych* OR “mental health”)	
EMBASE 1291 results	Title, Abstract, Keywords English, Portuguese, Spanish, from 1 January 2020 to 1 October 2023	1	SARS-CoV-2 OR coronavirus OR COVID-19 OR pandemic	1 AND (2 AND 3) AND (4 AND 5)
		2	vaccin* OR immunization OR mask OR quarantine OR lockdown OR ‘social distancing’ OR ‘social isolation’ OR isolation OR hygiene	
		3	hesitan* OR accept* OR reject* OR uptake OR adheren* OR refus*	
		4	patient* OR client*	
		5	psych* OR “mental health”	
WEB OF SCIENCE 382 results	Topic Field English, Portuguese, Spanish, from 1 January 2020 to 1 October 2023	1	SARS-CoV-2 OR coronavirus OR COVID-19 OR pandemic	1 AND 2 AND 3
		2	(vaccin* OR immunization OR mask OR quarantine OR lockdown OR ‘social distancing’ OR ‘social isolation’ OR isolation OR hygiene) AND (hesitan* OR accept* OR reject* OR uptake OR adheren* OR refus*)	
		3	(patient* OR client*) AND (psych* OR “mental health”)	
PsycINFO 109 results	Title, Abstract, Keywords From 2020 to 2023	1	Title: SARS-CoV-2 OR coronavirus OR COVID-19 OR pandemic	(1 AND 2 AND 3) OR (4 AND 5 AND 6) OR (7 AND 8 AND 9)
		2	Title: (vaccin* OR immunization OR mask OR quarantine OR lockdown OR ‘social distancing’ OR ‘social isolation’ OR isolation OR hygiene) AND (hesitan* OR accept* OR reject* OR uptake OR adheren* OR refus*)	
		3	Title: (patient* OR client*) AND (psych* OR “mental health”)	
		4	Abstract: SARS-CoV-2 OR coronavirus OR COVID-19 OR pandemic	
		5	Abstract: (vaccin* OR immunization OR mask OR quarantine OR lockdown OR ‘social distancing’ OR ‘social isolation’ OR isolation OR hygiene) AND (hesitan* OR accept* OR reject* OR uptake OR adheren* OR refus*)	
		6	Abstract: (patient* OR client*) AND (psych* OR “mental health”)	
		7	Keywords: SARS-CoV-2 OR coronavirus OR COVID-19 OR pandemic	
		8	Keywords: (vaccin* OR immunization OR mask OR quarantine OR lockdown OR ‘social distancing’ OR ‘social isolation’ OR isolation OR hygiene) AND (hesitan* OR accept* OR reject* OR uptake OR adheren* OR refus*)	
		9	Keywords: (patient* OR client*) AND (psych* OR “mental health”)	

**Table 2:** Summary of qualitative studies.

No.	First Author and Publication Year	Country of Origin	Study Design	Recruitment Setting and Sample Size	Patients’ Psychiatric Diagnoses	Preventive Behavior Adherence Across Groups
**1**	Kotlarska, 2021 [23]	Poland	Qualitative study	10 outpatients from the Babinski Hospital in Krakow	SCZ	- All patients stated that they followed most or all temporary pandemic restrictions. However, their attitudes toward these limitations varied. Five patients found no issues with adhering to the restrictions, while the other five believed that the restrictions were excessive or noticed negative impacts on their relationships or overall mental well-being.
**2**	Clingan, 2023 [24]	US	Qualitative study	32 outpatients from 15 SUD specialty treatment centers, a federally qualified health center and medication treatment for OUD providers in Southern California	OUD	- Participants held mixed beliefs regarding their susceptibility to contracting COVID-19 and its severity. Some took precautions to prevent infection, while others prioritized substance use over COVID-19 concerns. - Most received at least one COVID-19 vaccine, yet half expressed hesitancy. Concerns included unknown long-term side effects and potential interactions with medication for OUD.
**3**	Grove, 2023 [25]	US	Qualitative study	50 outpatients from a Texas community mental health center	SMI	- Vaccine concerns affected nearly half of the patients, including worries about rapid development, FDA approval, side effects, doubts about effectiveness, and conspiracy-based beliefs. - Less than half of the participants discussed the vaccine with a healthcare provider, with many decisions influenced by friends and family. - Convenient vaccination circumstances, such as being offered by healthcare providers or at familiar locations, facilitated uptake.

Note: FDA: Food and Drug Administration; OUD: opioid use disorder; SCZ: schizophrenia; SMI: severe mental illness; SUD: substance use disorder.

**Table 3:** Summary of cross-sectional studies with mixed samples.

No.	First Author and Publication Year	Country of Origin	Study Design	Recruitment Setting and Sample Size	Patients’ Psychiatric Diagnoses	Preventive Behavior Adherence Across Groups
**1**	Hao, 2021 [26]	China	Cross-sectional	79 outpatients from the psychiatric clinics of five hospitals in Chongqing, China	Mixed	- Patients showed high acceptance and willingness to pay for the COVID-19 vaccine. - People with MDD or anxiety disorder were more willing to pay for the COVID-19 vaccine than healthy controls, and severity of symptoms was the most important associated factor. - No significant differences were found between individuals with MDD or anxiety disorders and healthy controls regarding safety concerns over vaccines, willingness to receive other vaccines, and outlook on vaccines leading to a return to normal life.
**2**	Huang, 2021 [27]	China	Cross-sectional	906 patients from the Wuhan Mental Health Center	Mixed	- Compared to the general population, patients showed slightly lower VA and relatively higher hesitancy. - A large gap between vaccine acceptance and uptake rates among patients was observed, indicating that other vaccination barriers specific to mental disorders may complicate vaccine uptake. - Factors associated with VA: perceiving a high risk of death, having reduced income due to the COVID-19 pandemic, perceiving a good preventive effect of vaccines, believing that ≥50% of vaccine recipients would be immune, believing that vaccines are safe, and having good insight into mental disorders. - Factors associated with VH: controversy regarding the priority of patients with mental disorders, stigma surrounding mental disorders, impaired decision-making ability, and lack of clinical guidelines for vaccination in individuals with mental disorders. - Persons with psychotic disorders were least likely to take the vaccine.
**3**	Jefsen, 2021 [28]	Denmark	Cross-sectional	608 patients from psychiatric services of the Central Denmark Region	Mixed	- Vaccine willingness was slightly lower among people with mental illness compared with the general population; however, VH does not appear to be a major barrier to vaccine uptake amongst patients. - Across both groups, younger age was strongly associated with hesitancy. For patients, divorce or repeal of the registered partnership was also positively associated with VH. - No psychiatric diagnosis was particularly strongly associated with VH.
**4**	Mazereel, 2021 [29]	Belgium	Cross-sectional	1151 patients from a university psychiatric hospital in Belgium	Mixed	- A high VR was found among the patients, similar to the general population in Flanders. - No effect of diagnosis on vaccination status was observed.
**5**	Ren, 2021 [30]	China	Cross-sectional	408 subjects from the Tongde Hospital of Zhejiang Province, Hangzhou, China	Mixed	- A high level of VA was observed among the sample, and among those who would decline vaccination, vaccine safety was the main concern. - Over half expressed that they were more likely to vaccinate against COVID-19 if doctors or disease control personnel suggested this option to them.
**6**	Uvais, 2021 [31]	India	Cross-sectional	90 outpatients from a tertiary care hospital in Kerala, India	Mixed	- Half of the patients reported a definitive or probable intention to receive COVID-19 vaccination, while one-third reported a definitive intention not to receive vaccination. COVID-19 VH was significantly higher compared to studies conducted among the general population across the world. - There was no significant difference in vaccination intention between patients with neurotic spectrum disorder and psychotic spectrum disorder.
**7**	Ateş, 2023 [32]	Türkiye	Cross-sectional	212 patients from the Child and Adolescent Psychiatry Outpatient Clinic of Gülhane Training and Research Hospital	Mixed	- The VR was higher in patients with anxiety disorders. - Age, parental VH, parental vaccination status, and chronic disease in a family member can affect the vaccination of adolescents.
**8**	Eyllon, 2022 [33]	USA	Cross-sectional	14,365 subjects from a northeastern multi-specialty group medical practice, of which 42.4% were outpatients with psychiatric comorbidities	Mixed	- VH was more prevalent across all psychiatric comorbidities, except for AUD, with a lower rate among anxiety disorders and higher rate in SUD. - Slightly greater proportions of patients with MDD, BD and GAD reported vaccine resistance compared to reference groups.
**9**	Mashima, 2022 [34]	Japan	Cross-sectional	1166 outpatients from a psychiatric hospital and a mental health clinic in Chiba, Japan	Mixed	- No significant difference was found in the items regarding adaptation to daily life during the pandemic, and the majority reported that they are already used to their daily life. - Among the mood disorder group, a significant decrease in time spent outside compared to patients in other diagnostic groups was observed, while in the psychotic diagnosis group, patients engaged in online shopping and attended online meetings less frequently than patients in other diagnostic categories. Additionally, they wore a mask, changed their mask, or washed their mask less frequently than other groups. - Most patients always washed their hands and gargled, but this was less frequent in the psychotic disorder group, which also showed poorer hygiene. The group with neurotic, stress-related, and somatoform disorders showed a better hygiene score.
**10**	McNeil, 2022 [35]	Canada	Cross-sectional	96 outpatients from the Anxiety Studies database at the University of Waterloo	Mixed	- There were no significant differences in VH or motivation to get vaccinated between people with anxiety disorders and those without. - The same factors that predict VH for well-known vaccines also predict COVID-19 VH for both anxious and non-anxious people. - Intolerance of uncertainty was a significant unique predictor of VH, with higher IUS associated with greater hesitancy.
**11**	Qin, 2022 [36]	China	Cross-sectional	2250 subjects from the psychiatric outpatient and inpatient department of the Second Xiangya Hospital of Central South University, China, of which 1328 were psychiatric outpatients and 922 were family members	Mixed	- A lower VR was observed among patients compared to their family members. - The VR was higher in psychiatric patients aged 18–34, those with higher education levels, and urban residents. - Patients with schizophrenia showed a lower VR than those with other psychiatric disorders, and hospitalized individuals also had lower VR than outpatients. - Patients reported concerns about effectiveness and safety, exacerbating psychiatric disorders and exacerbating side effects of psychotropic drugs.
**12**	Pisl, 2023 [37]	Czech Republic	Cross-sectional	6,757,155 subjects from medical records from the Institute of Health Information and Statistics of the Czech Republic	Mixed	- Compared to the general population, mental healthcare users were under-vaccinated in August but not in December 2021. - Vaccine uptake was lower in those with a history of psychiatric hospitalizations but higher in those who had recently utilized inpatient or outpatient mental healthcare, predominantly for affective disorders.
**13**	Ryu, 2023 [38]	Republic of Korea	Cross-sectional	572 outpatients from two university hospitals, two mental hospitals, and two community mental health centers in South Korea	Mixed	- Individuals in the high-VA group were older, more likely to receive the influenza vaccine regularly, and more likely to trust formal information sources. They had higher levels of gratitude and agreeableness, and lower levels of depression and neuroticism.

Note: AUD: alcohol use disorder; BD: bipolar disorder; GAD: generalized anxiety disorder; IUS: intolerance of uncertainty scale; MDD: major depressive disorder; SUD: substance use disorder; VA: vaccination acceptance; VH: vaccination hesitancy; VR: vaccination rate.

**Table 4:** Summary of cross-sectional studies with SMI samples.

No.	First Author and Publication Year	Country of Origin	Study Design	Recruitment Setting and Sample Size	Patients’ Psychiatric Diagnoses	Preventive Behavior Adherence Across Groups
**1**	Muruganandam, 2020 [39]	India	Cross-sectional	132 outpatients from a tertiary general hospital in South India	SMI	- Nearly three-fourths of patients with SMI did not have adequate knowledge about symptoms and preventive measures in the context of COVID-19. Among them, around 8% were completely unaware of the ongoing pandemic. - Younger age, greater educational attainment, higher socioeconomic status, and increased levels of perceived social support were associated with significantly higher knowledge scores in the sample.
**2**	Bitan, 2021 [40]	Israel	Cross-sectional	50,240 subjects from Clalit Health Services database, of which 50% were patients with SCZ	SCZ	- The odds of receiving COVID-19 vaccination were significantly lower in the schizophrenia group compared to the control group. - Differences were more profound as age increased (especially >60), and in the 16–21 age subsample, no significant differences were observed. - The odds of being vaccinated were lower in the SCZ group for both male and female participants, with males showing slightly greater gaps in VR.
**3**	Hoşgelen, 2021 [41]	Turkey	Cross-sectional	76 patients from the Psychosis Outpatient Unit of Dokuz Eylul University Hospital Psychiatry Department	SCZ	- A minority of patients reported wearing masks in public settings, whereas the majority mentioned adhering to social distancing measures.
**4**	Macdonald, 2021 [42]	US	Cross-sectional	57 patients from mental health clinics and the Georgia Psychiatric Risk Evaluation Program	SCZ and individuals at CHR for psychosis	- For CHR patients, fear of COVID-19 hindered enjoyment of activities and socializing more than in community controls, while SCZ diagnoses were linked to adherence to health/safety guidelines.
**5**	Masson, 2021 [43]	US	Cross-sectional	265 outpatients from 11 residential SUD treatment programs in California	SMI	- Most participants were aware of COVID-19 modes of transmission, but around one-third trusted that a COVID-19 vaccine would be safe and effective. - Factors independently associated with trust in a COVID-19 vaccine included increased age and wearing a mask all the time.
**6**	Pinkham, 2021 [44]	US	Cross-sectional	163 outpatients from the National Institute of Mental Health-funded studies (the University of Texas at Dallas, the University of California, San Diego, and the University of Miami)	SMI	- Individuals with SMI did not significantly differ from healthy controls in terms of self-distancing, avoiding public spaces/transportation, avoiding in-person visits, staying home, handwashing/using sanitizer, and cleaning. A greater percentage of individuals with SMI reported wearing a face mask, which was marginally statistically significant. - Individuals with affective disorders were more likely to use face masks, to stay home, to work remotely and to avoid public spaces. - Engaging in self-distancing was significantly associated with increased educational achievement. Wearing a face mask was significantly associated with residential status, with higher proportions among those living independently or in supervised settings, and was also linked to lower negative symptom severity at baseline. Avoiding in-person visits was related to increased loneliness and better coping, and staying home was associated with increased anxiety.
**7**	Bai, 2021 [45]	China, US, Australia	Cross-sectional	1853 patients from clinics for community-dwelling patients and inpatient departments attached to psychiatric hospitals in China	SMI	- There was no significant difference in VH across MDD, BD, and SCZ subgroups. - Patients with a higher level of perceived stigma were more likely to have VH.
**8**	Cai, 2022 [46]	China	Cross-sectional	1149 out- and inpatients from six major psychiatric hospitals in China	SMI	- Over half of patients with depressive disorder expressed willingness to receive a COVID-19 vaccine, a lower rate than in non-clinical adult samples. - Those who accepted future vaccinations were significantly less likely to report suicidality in the past year, to report severe physical pain, and to report depression symptoms than those who hesitate.
**9**	Haderlein, 2022 [47]	US	Cross-sectional	4,890,693 subjects from Veterans/VHA Corporate Data Warehouse, of which 4% had SMI	SMI	- Nearly half of veterans diagnosed with SMI received a COVID-19 vaccination, which was similar to the VR among those without SMI. The impact of SMI on the likelihood of COVID-19 vaccination was not statistically significant.
**10**	Jia, 2022 [48]	China	Cross-sectional	444 inpatients from Tianjin Jianhua Hospital	SCZ	- Most patients accepted the vaccine, and vaccination refusal was related to the higher baseline level of psychotic symptoms. - Possible factors related to VH in patients include impaired judgment, decreased foresight, low educational level, and fear or anxiety, which are all correlated with the severity of psychiatric symptoms.
**11**	Walton, 2022 [49]	Australia	Cross-sectional	46 patients from the Peel and Rockingham Kwinana Mental Health Service in Western Australia	Early episode psychosis	- Around half of the patients intended to get vaccinated, while half were worried about side effects, and one-third believed that vaccines were safe. The findings suggest that the VR among those with psychotic illnesses is lower than the federal targets. - Many participants believed that more research, information, and improved accessibility would assist them.
**12**	Raffard, 2022 (A) [50]	France	Cross-sectional	100 outpatients from CHU Montpellier, CHU Grenoble, CHU Versailles and AP-HP Marseille	SCZ	- The study found lower VR in patients compared to controls. - Vaccination status was not significantly associated with global cognitive functioning, psychotic symptoms, or paranoid ideations. - Patients reported higher confidence in the benefits of vaccination compared to controls. - The preference for the natural immunity VAX dimension was related to higher levels of negative psychotic symptoms and higher levels of paranoid ideation.
**13**	Raffard, 2022 (B) [51]	France	Cross-sectional	80 outpatients from the University Department of Adult Psychiatry of Montpellier	SCZ	- A lower VR was observed among the patients compared to the general French population. - Over half of the sample was classified as having diminished capacity to consent to the vaccination, and this was associated with lower VR, poorer cognition, and higher levels of psychotic symptoms.
**14**	Uvais, 2022 [52]	India	Cross-sectional	62 patients from 7 Community Psychiatry Clinics in Northern Kerala	SMI	- A significantly lower VR was observed among the patients compared to the general population, and around half were recommended for vaccination by a healthcare provider. - The recommendation of vaccination by healthcare providers was a significant modifiable factor influencing vaccination status among individuals with SMI.
**15**	Hooper, 2023 [53]	Canada	Cross-sectional	142 inpatients from the MHU at a Western Australia tertiary institution using notes and online national health databases	SMI	- Patients’ VR was significantly lower than the regional average in January 2022, but this difference decreased over time. - Over half of the patients accepted vaccination when offered, representing a significant potential for targeted programs to improve VR in the SMI population. - The most common reasons for refusal were delusional thoughts, concerns regarding side effects or lack of long-term safety data, and ambivalence towards the risk from COVID-19.

Note: BD: bipolar disorder; CHR: clinical high risk; CHU: Centre Hospitalier Universitaire; MDD: major depressive disorder; MHU: mental health unit; SCZ: schizophrenia; SMI: severe mental illness; SUD: substance use disorder; VAX: vaccine; VH: vaccination hesitancy; VR: vaccination rate.

**Table 5:** Summary of cross-sectional studies with samples of neurodevelopmental disorders.

No.	First Author and Publication Year	Country of Origin	Study Design	Recruitment Setting and Sample Size	Patients’ Psychiatric Diagnoses	Preventive Behavior Adherence Across Groups
**1**	Mutluer, 2020 [54]	Turkey	Cross-sectional	87 patients from the Koc University Hospital—child psychiatry outpatient unit database	ASD	- Most patients in the study faced difficulties understanding COVID-19, adhering to social distancing and stay-at-home guidelines, and performing necessary hygiene practices. Individuals with ASD commonly encounter challenges such as hypersensitivity, which can impede mask-wearing and limit their ability to take precautions effectively.
**2**	Weinstein, 2021 [55]	Israel	Cross-sectional	5540 ASD outpatients from the Clalit Health Services database	ASD	- Individuals with ASD were more likely to be vaccinated for COVID-19 compared to the general population across both sexes, but only in the 16–20 and 21–40 age groups.
**3**	Kilic, 2022 [56]	Turkey, United Kingdom, Iceland	Cross-sectional	90 patients from the psychiatry outpatient clinic of a tertiary university hospital	ADHD	- Similar rates were found between adults with ADHD and controls in terms of COVID-19 infection status, vaccination adherence, perception of COVID-19 as dangerous, and “behavioral avoidance” towards COVID-19. - Patients exhibited a greater perception of COVID-19 as contagious, a positive attitude toward the vaccine, and “cognitive avoidance” of COVID-19 compared to controls. - Clinical characteristics of ADHD symptoms, state and trait anxiety, and depression did not play a significant role in the observed outcomes.
**4**	Zemer, 2023 [57]	Israel	Cross-sectional	46,544 subjects from Clalit Health Services database—Dan-Petach-Tikva, of which 17.7% were outpatients with ADHD	ADHD	- Adolescents with ADHD were less prone to be infected with COVID-19, and older adolescents were more likely to be vaccinated. - The population of Jewish adolescents with ADHD achieved higher COVID-19 VR compared to their non-ADHD counterparts.
**5**	Khoodoruth, 2023 [58]	Qatar	Cross-sectional	243 parents or guardians of ASD outpatients from the Pediatric Emergency Center	ASD	- The COVID-19 VR did not differ between the ASD group and controls. - Among parents or caregivers of children with ASD, approximately two-thirds refused or were unsure about vaccinating their children.

Note: ADHD: attention deficit hyperactivity disorder; ASD: autism spectrum disorder; VR: vaccination rate.

**Table 6:** Summary of longitudinal studies.

No.	First Author and Publication Year	Country of Origin	Study Design	Recruitment Setting and Sample Size	Patients’ Psychiatric Diagnoses	Preventive Behavior Adherence Across Groups
**1**	Bitan, 2021 [59]	Israel	Longitudinal retrospective	51,078 subjects from Clalit Health Services database, of which 50% were patients with SCZ	SCZ	- The control group showed a sharper incline in the probability of vaccination. - Medical comorbidity of diabetes, hypertension, obesity, or ischemic heart disease played a significant role in predicting VR in the schizophrenia group, but not in the control group.
**2**	Danenberg, 2021 [60]	Israel	Longitudinal retrospective	51 patients from the Shalvata Mental Health Center	Mixed	- The majority of the sample were vaccinated. - Fear of COVID-19 levels did not differ between vaccinated and unvaccinated individuals, but vaccinated patients scored higher on the COVID-19 Vaccine Hesitation Scale, indicating a positive attitude towards vaccination. - Patients’ decisions are influenced by their viewpoints, similar to the general population. - Having psychotic, bipolar, or personality spectrum disorders did not predict attitudes toward vaccination.
**3**	Mazereel, 2021 [61]	Belgium	Longitudinal retrospective	2105 inpatients from the University Psychiatric Center KU Leuven	Mixed	- The total VR in the study was similar to the general population. - Type of disorder does not seem to play a role in the VR, although ‘other diagnoses’ were less likely to be vaccinated. This category mostly consists of cases of deferred diagnosis or psychosocial problems. - An effect of age (with older people being more likely to be vaccinated) and residing in the psychosocial center on vaccination status was observed.
**4**	Moeller, 2021 [62]	US	Longitudinal retrospective	174 inpatients from a child and adolescent psychiatric hospital of the University of Kansas Health System in Marillac campus	Mixed	- Over half of the patients were unvaccinated, and among them, most declined vaccination. - The study found an acceptance rate in adolescent inpatients with mental illness comparable to the national average in June 2021. However, it is significantly lower than the intention rate reported in a survey in April 2021. - The main reasons for VH were concern over safety and efficacy from parents.
**5**	Sánchez-Guarnido, 2021 [63]	Spain	Longitudinal retrospective	270 subjects from 15 mental health day hospitals (MHDHs) of the Spanish National Health System, of which 185 had SMI	SMI vs. other disorders	- The mask use rate between groups was similar, while the rates of social distancing and handwashing were significantly lower in the SMI group.
**6**	Barone, 2022 [64]	Italy	Longitudinal prospective	114 patients from the Psychiatric Unit of the University of Naples “Federico II”	SMI	- There were four times more non-vaccinated individuals in the patient group than in the control group. - Higher paranoia mean scores were observed in vaccine-hesitant patients compared to vaccinated patients.
**7**	Bitan, 2022 [65]	Israel	Longitudinal retrospective	69,594 subjects from Clalit Health Services database, of which 50% were patients with SCZ.	SCZ	- Individuals with schizophrenia were less likely to be vaccinated with the COVID-19 booster vaccine, and gaps in vaccination were largest for the first vaccination. - Time to reach vaccination was significantly longer for the SCZ group but primarily with the first vaccine and to a smaller extent with the booster vaccine.
**8**	Hassan, 2022 [66]	UK	Longitudinal retrospective	230,567 patients from the electronic health record data from the Greater Manchester Care Record (GMCR)	SMI vs. other disorders	- People with SMI, particularly with mood disorders, were significantly more likely to be vaccinated than people without SMI. Despite this, individuals with schizophrenia or related psychotic disorders were significantly more likely to have a record of having declined vaccination for COVID-19. - Compared to matched controls, VR was highest among people with RDD, followed by BD, other depressive disorders, and psychotic disorders.
**9**	Korkmaz, 2022 [67]	Turkey	Longitudinal retrospective	120 patients from the Psychiatry Outpatient Clinic of Fırat University	Mixed	- There were similar rates for total cleanliness, social distancing, and hygiene scores in the groups, despite the higher health anxiety level in the control group. The mask-wearing scores for the patient group were higher compared to the control group, while food and beverage hygiene scores were higher in the control group. - The highest adherence scores in both groups were for hand hygiene, ventilation of the room, and avoidance of indoor spaces, and the lowest adherence in both groups was for social distancing. - The scores of women in the patient group were higher, and they adhered to the rules more than the men.
**10**	Shaffer, 2022 [68]	US	Longitudinal retrospective	12,714 subjects from a tertiary care academic medical center and a freestanding psychiatric hospital in New York, of which 1029 were psychiatry patients and 11,685 were medical and surgical patients	Mixed	- Patients admitted to psychiatric services had a significantly lower VR than patients admitted to the medical and surgical facility. Moreover, VR among medical and surgical inpatients increased every month during the study, while among psychiatric inpatients, it never exceeded 50% during the study.

Note: BD: bipolar disorder; RDD: recurrent depressive disorder; SCZ: schizophrenia; SMI: severe mental illness; VH: vaccination hesitancy; VR: vaccination rate.

**Table 7:** Summary of interventional studies.

No.	First Author and Publication Year	Country of Origin	Study Design	Recruitment Setting and Sample Size	Patients’ Psychiatric Diagnoses	Preventive Behavior Adherence Across Groups
**1**	Lim, 2022 [69]	US	Longitudinal prospective and interventional	193 patients from an outpatient clozapine treatment clinic housed in a community mental health center in Boston	SMI	- A high VR was observed among patients compared to the rate in Massachusetts, and the VH of the sample was similar to that of the broader US population. - The reasons for VH were adverse effects and safety of vaccines, belief that COVID-19 was not a serious threat or that vaccines were unnecessary, belief in immunization after contracting COVID-19, mistrust of government and pharmaceutical companies, or endorsed conspiracy theories. - Proactive intervention by psychiatric providers who addressed COVID-19 vaccine-related concerns and encouraged vaccinations at routine visits resulted in higher VR among patients.
**2**	Peritogiannis, 2022 [70]	Greece	Longitudinal prospective and interventional	197 outpatients from 2 community mental health services (MMHUs) in remote areas in Greek islands	SMI	- The VR in the sample with SMI did not differ from the general population. - The most common reasons for VH are also encountered in the general population, including fears, concerns, and false beliefs, whereas one-third of the refusal had been associated with symptomatology, mostly positive and cognitive symptoms. - There were no differences in VR concerning the interventions that were applied to enhance vaccination, nor among patients with different diagnoses.
**3**	Venegas-Murillo, 2022 [71]	US	Longitudinal prospective and interventional	180 subjects from an academic–community partnered collaboration between Charles R. Drew University of Medicine and Science and Tessie Cleveland Community Services Corporation in Los Angeles and Riverside Counties.	Mixed	- Post-test was associated with an increase in vaccination uptake that indicated the efficacy of the intervention in promoting vaccination and reducing VH. - The results showed that community-based mental health therapists play a pivotal role in mitigating COVID-19 risk and VH among their clients.
**4**	Veltro, 2022 [72]	Italy	Longitudinal prospective and interventional	37 patients from a community mental health center	SMI	- Most of the sample accepted the vaccine. - The results suggest that the applied psychoeducational intervention positively influences SMI patients during the pandemic, leading to improved personal and social functioning and increased self-determination regarding vaccination.

Note: MMHU: mobile mental health unit; SMI: severe mental illness; VH: vaccination hesitancy; VR: vaccination rate.

## Data Availability

No new data were created or analyzed in this study. All data analyzed during this study are included in this published article.

## Supplementary Materials

The following supporting information can be downloaded at https://www.mdpi.com/article/10.3390/ijerph23081001/s1. Table S1: PRISMA-ScR checklist of this scoping review. Ref. [22] was cited in the Supplementary Material.
